# Supplementary material for: iModulonMiner and PyModulon: Software for unsupervised mining of gene expression compendia
Source: PLoS Comput Biol. 2024 Oct 23;20(10):e1012546. doi: 10.1371/journal.pcbi.1012546 (PMC11534266; doi:10.1371/journal.pcbi.1012546)
Supplement: S1 Text — Supplementary Information file that contains Supplementary Methods, Results, Notes A-E, Figs A-K and References. (PDF) [file pcbi.1012546.s001.pdf]

Supplementary Information for:

iModulonMiner and PyModulon: Software for unsupervised mining of gene expression compendia

Supplementary Methods

Manual annotation of experimental metadata

Inaccurate or insufficient metadata reporting can hinder the widespread utilization of public data and potentially prevent subsequent interpretation [1,2]. Therefore, manual metadata curation is typically performed on the data that pass other quality control steps. Information including the strain description, base media, carbon source, treatments, temperature and the growth stage (e.g., mid-exponential, stationary, biofilm, etc.) were pulled from the literature if they were reported. Each project was assigned a short unique name, and each condition within a project was also assigned a unique name to identify biological and technical replicates. After curation, samples were discarded if (a) metadata was not available, (b) samples did not have replicates, or (c) the Pearson R correlation between replicates was below 0.95.

Computing iModulon thresholds

Each independent component contains the contributions of each gene to the statistically independent source of variation. Most of these values are near zero for a given component. In order to identify the most significant genes in each component, we iteratively removed genes with the largest absolute value and computed the D’Agostino K2 test statistic [3] for the resulting distribution. Once the test statistic dropped below a cutoff, we designated the removed genes as significant.

To identify this cutoff, we performed a sensitivity analysis on the concordance between significant genes in each component and known regulons. First, we isolated the 20 genes from each component with the highest absolute gene coefficients. We then compared each gene set against all known regulons using the two-sided Fisher’s exact test ($FDR < 10^{-5}$). For each component with at least one significant enrichment, we selected the regulator with the lowest p-value.

Next, we varied the D’Agostino K2 test statistic from 50 through 2000 in increments of 50, and computed the F1-score (harmonic average between precision and recall) between each component and its linked regulator. The maximum value of the average F1-score across the

components with linked regulators occurred at a test statistic of cutoff of 450 for the *B. subtilis* dataset.

For future datasets where a draft TRN is unavailable, an alternative method is proposed that is agnostic to regulator enrichments. The Sci-kit learn [4] implementation of K-means clustering, using three clusters, can be applied to the absolute values of the gene weights in each independent component. All genes in the top two clusters are deemed significant genes in the iModulon.

Compiling gene annotations

The gene annotation pipeline can be found at https://github.com/SBRG/pymodulon/blob/master/docs/tutorials/creating_the_gene_table.ipynb. Gene annotations for Bacillus subtilis strain 168 were pulled from the NCBI database with accession AL009126.3. Additionally, KEGG [5] and Cluster of Orthologous Groups (COG) information were obtained using EggNOG mapper [6]. Uniprot IDs were obtained using the Uniprot ID mapper [7], and operon information was obtained from Biocyc [8]. Gene ontology (GO) annotations were obtained from AmiGO2 [9]. The known TRN was obtained from *Subti*Wiki [10].

Computing iModulon enrichments

iModulon enrichments against known regulons were computed using Fisher's Exact Test, with the false discovery rate (FDR) controlled at 10^{-5} using the Benjamini-Hochberg correction. Fisher's Exact Test was used to identify GO and KEGG annotations as well, with an FDR < 0.01.

Additional functions for gene set enrichment analysis are located in the *enrichment* module, including a generalized gene set enrichment function and an implementation of the Bonferroni-Hochberg false discovery rate (FDR).

Motif search

Motif searches were performed using the *find_motif* function in PyModulon, which is a wrapper for MEME [11]. For each operon in an iModulon we searched for motifs in the sequence ranging from 500 base pairs upstream to 100 base pairs downstream of the gene start. For each iModulon, we searched for five motifs with zero or one occurrence per sequence, with an E-value threshold of 0.001. All motif widths were examined between 6 and 40 base pairs.

iModulon motifs were compared to known motifs using the *compare_motifs* function in PyModulon, which is a wrapper for TOMTOM [11] using an E-value of 0.001. Motifs were compared against five prokaryotic motif databases [12–16].

Clustering iModulon activities

Global iModulon activity clustering was performed using the *clustermap* function in the Python Seaborn package [17] using the following distance metric:

$$d_{x,y} = 1 - ||\rho_{x,y}||$$

where $||\rho_{x,y}||$ is the absolute value of the Spearman R correlation between two iModulon activity profiles. The threshold for optimal clustering was determined by testing different distance thresholds to locate the maximum silhouette score (**Figure F Panel C**).

Estimating iModulon activities for external datasets

The dataset for biofilm development was downloaded from NCBI GEO (GSE141305) and processed using the previously described pipeline. Data was centered on the reference condition (liquid culture). To infer the iModulon activities, the following equation was used:

$$\mathbf{A'} = \mathbf{M}^{-1} \cdot \mathbf{X'},$$

where **A'** is the inferred activity matrix, $\mathbf{M}^{-1}$ is the pseudo-inverse of the precomputed independent components from the *B. subtilis* compendium, and **X'** is the expression matrix of the new data.

Comparing iModulon structures

To compare iModulon structures between datasets from the same organism, each iModulon from one dataset was compared against every iModulon in the other dataset using the following distance metric:

$$d_{x,y} = 1 - ||\rho_{x,y}||$$

where $||\rho_{x,y}||$ is the absolute value of the Pearson R correlation between two independent components. iModulons with distances less than 0.25 were considered identical.

Before comparing iModulon structures across different organisms, gene orthology must be determined. As a simple metric, we used reciprocal BLAST hits to generate a one-to-one orthology between *E. coli* and *B. subtilis*. Once the orthologous pairs were determined, the iModulons were compared as described above.

Creating an iModulonDB web page

Since the iModulon decomposition for an organism is a knowledge-base that will be of interest to a broad audience of microbiologists, we created an interactive website (iModulonDB.org) for researchers to explore published iModulons [18]. iModulonDB lists all iModulons identified for a dataset, which link to interactive dashboards for each iModulon. Users can explore an

iModulon's member genes, activity levels across conditions, and comparisons with known regulons. Each gene also has its own page, with links to external databases, strengths of relationships to iModulons, and an expression profile. Within a dataset, users can search for genes or regulators of interest and browse all iModulons to obtain co-regulated gene sets or insights about condition-dependent activity.

The function *imodulondb_export* automatically generates all relevant files for an organism's iModulonDB website from an *IcaData* object. Automated error checking and compatibility with the site javascript is ensured by the function *imodulondb_compatibility*. While these functions will work with a minimally curated *IcaData* object, the site experience is improved by the addition of hyperlinks for genes, transcription factors, metadata, and details about each sample. A Jupyter notebook [19] guiding the addition of these annotations can be found at the following link (https://pymodulon.readthedocs.io/en/latest/tutorials/creating_an_imodulondb_dashboard.html).

Although the public iModulonDB site only contains our published decompositions, users can download its content from the github repository (https://github.com/SBRG/modulytics), add the files for their own decomposition, and then host a local version of the site with their data. Instructions for doing so are available in the iModulonDB github wiki (https://github.com/SBRG/modulytics/wiki/Adding-a-Private-Project). This method ensures privacy for new data while still enabling the search and dashboard utility of iModulonDB.

Creating a draft experimental TRN

It is recommended to compile a draft TRN, as it will be used to associate computed iModulons with known regulators. For model organisms, such as *B. subtilis* and *E. coli*, dedicated databases already exist with this information [10,20]. Although some resources are available for less-characterized organisms [21,22], most information must be scraped from primary literature.

Supplementary Results

Estimating regulatory activities for new datasets using established iModulons

Recomputing the full set of iModulons can be computationally intensive for each new dataset, particularly when the quality of the new data is uncertain or a rapid assessment is required. The *infer_activities* function leverage a pre-computed *IcaData* object to infer the iModulon activity for the new dataset without the need for a complete recomputation.

To demonstrate this functionality, we downloaded and processed an RNA-seq dataset tracking the development of *B. subtilis* biofilms [23]. This dataset was published after we compiled the transcriptomic compendium, so it was not used to compute the *B. subtilis* iModulon structure.

The resulting activity matrix (**Figure H**) clearly shows that the sigma factors SigK and SigE are highly active during intermediate biofilm formation, between 2 days and 14 days, as compared to the reference condition of a fresh liquid culture. This likely explains the observation in the original study that the expression of transcription sigma factors increased during the intermediate biofilm stage [23]. The three iModulons related to the stress response sigma factor SigB exhibit an initial drop in activity but return to the reference levels after a day. iModulons related to carbon metabolism are generally down-regulated across the biofilm time-course, whereas iModulons encoding the PBSX phage (Xre/Xpf iModulon), capsular polysaccharide (EAR riboswitch), and competence (Rok) are up-regulated. Altogether, iModulons provide a fine-grained, while systems-level, view of the transcriptional changes occurring during biofilm formation.

Applying ICA to eukaryotic datasets: Methods and results overview

Selection of 131 samples of publicly available data for *S. cerevisiae* strain CEN.PK was performed for analysis in order to display the potential of iModulon analysis to generate biological discoveries in eukaryotic organisms. Samples were aligned and quality control was performed based on described workflow steps and alternatives. ICA optimal dimensionality selection revealed 38 robust components explaining 85% of the variance in the dataset (**Figure J Panel A**), indicating accuracy of numerical reconstruction of the original data in line with iModulon studies in prokaryotic organisms. Analysis of component 17 revealed coordination of genes involved in chromatin dynamics of the cell being positively regulated (HTB2 and HTA2) while proteins related to cell wall stability (HSP150) were downregulated. The activity pattern of this iModulon displays an increase in conditions related to cell S-phase in data associated with coordinated cell cycles in CEN.PK (**Figure J**). Cyclical activation of this component displays coordinated activity of these genes in relation to cell state and stage of cell cycle. These findings parallel gene expression in the original publication and display the ability of iModulons to find biologically relevant components in *S. cerevisiae* [24].

Supplementary Notes

Supplementary Note A - ICA results on normalized count data

In many applications including differential expression analysis, it is typical to use normalized count data, such as that generated by DESeq2 (median of ratios) and TMM normalization, rather than TPM normalized data. We tested running ICA on the median of ratios-normalized count data (**Fig. A**). For bacteria, testing on E. coli in particular, we recovered nearly identical components compared to TPM normalized data, indicating that the workflow can handle either normalized count data or TPM normalized data equally well for prokaryotic applications. We expect that these differences will be more pronounced for eukaryotic data.

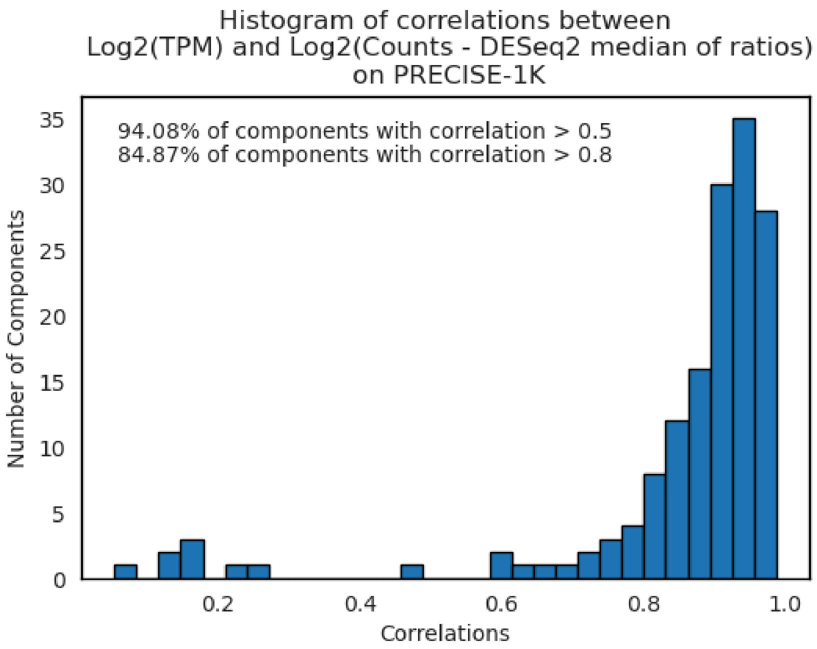

Figure A: Comparison of components obtained from running fastICA on TPM normalized vs normalized counts data.

Supplementary Note B - The M and A matrices

ICA is a matrix decomposition algorithm that takes a data matrix (**X**) and decomposes it into two smaller matrices (**M** and **A**), where **X** = **MA**. For this study, the **X** matrix contains a collection of gene expression profiles, where each column is a gene expression profile under a specific condition, and each row represents the expression of a single gene. Each column of the **M** matrix contains an independent component. Each independent component contains a weighting for each gene, and most gene weights in a component are near zero (**Figure 2b**). To identify genes with significant weightings, we apply a unique threshold to each component, and consider genes with weightings outside this threshold as part of the iModulon.

Each column of the A matrix contains the iModulon activities for a specific expression profile. Since we centered each expression profile to a project-specific reference condition, we can only compare activities across a project, rather than between projects. On the other hand, the gene weights represent the strength of regulation for a transcription factor on each gene. A gene with a high weight for a specific iModulon would be more sensitive to changes in iModulon activity than a gene with a low weight. Overall, the relative expression of a single gene under a specific condition is the sum of gene weights across all iModulons, each weighted by the conditions-specific iModulon activity (i.e. the product of the **M** and **A** matrices results in the expression matrix (**X**)).

Supplementary Note C - Computational performance at different dimensionalities

Time at different dimensionalities for the optICA calculation:

	100 FastICA runs (Total)	Distance Matrix & Clustering	Gathering final matrices
Dim 50	22.71 seconds	26.15 seconds	4.68 seconds
Dim 100	57.13 seconds	37.32 seconds	8.63 seconds
Dim 150	3.34 minutes	52.69 seconds	13.93 seconds
Dim 200	7.96 minutes	1.10 minutes	16.02 seconds
Dim 250	15.47 minutes	1.13 minutes	20.76 seconds
Dim 300	25.12 minutes	1.14 minutes	24.50 seconds

Resulting file sizes:

	Temporary files	Final M matrix	Final A matrix
Dim 50	543 MB	4.4 MB	970.8KB
Dim 100	1.1 GB	8.2 MB	1.8 MB
Dim 150	1.6 GB	11.4 MB	2.4 MB
Dim 200	2.2 GB	13.3 MB	2.9 MB
Dim 250	2.6 GB	15.4 MB	3.3 MB
Dim 300	3.3 GB	18 MB	3.9 MB

Supplementary Note D - The FastICA algorithm

FastICA is one of the most popular algorithms for ICA[25]. However, there are two considerations when running the FastICA algorithm. First, FastICA is a stochastic algorithm that results in different sets of independent components during different runs. Several solutions exist to identify components that are robust to random initialization[26,27]. In addition, one must set the number of components for ICA a priori. To address these issues, we previously published the optICA extension of the FastICA algorithm to identify robust components at the optimal dimensionality[28].

Briefly, optICA performs FastICA 100 times at a range of different dimensions. To identify the robust components at each dimension, the components are clustered to identify which components exist in at least 50% of the runs. These components comprise the ‘robust components.’ This process is repeated across a range of specified dimensions to track the number of robust components present at each dimension (**Figure B**). To detect under-decomposition, we also track the number of components at each dimension that are similar to the components found in the largest possible dimension (i.e. “final” components). To detect over-decomposition, we track the number of components that are dominated by a single gene (i.e. “single-gene” components). The optimal dimensionality exists where the number of “final” components overtakes the number of non-single-gene components. This optimal dimensionality is then used to identify the final iModulon set, which is composed of the component centroids of all DBSCAN clusters identified when running ICA 100 times at the optimal dimensionality.

Supplementary Note E - Analyzing iModulon differences across Datasets

As of early 2021, the NCBI SRA database contains over 55,000 microbial RNA-seq datasets. We have provided a computational workflow to convert this mass of raw data into a transcriptomic knowledgebase for each organism that connects observed expression changes to their underlying transcriptional regulators. The resulting iModulon structures can accelerate the discovery of new regulons, aid in the characterization of genes with unknown function, and enhance the analysis of new datasets. In addition, the processed RNA-seq data is a resource for further studies of global expression patterns for the organism.

We demonstrated the potential of this workflow by analyzing all publicly available high-quality RNA-seq datasets for *B. subtilis*. Through iModulons, we identified potential new targets for known transcription factors, and new regulons that require further investigation to discover their transcriptional regulator. In addition, investigation into an iModulon induced by the deletion of YonO, a single subunit phage RNA polymerase, led to the observation that heat shock likely disrupts the protein's function. The other iModulons capturing the SPβ prophage demonstrated that iModulons can provide understanding beyond just the TRN, and can be used to identify gene clusters such as prophages, pathogenicity islands, and biosynthetic gene clusters. Since the iModulons presented in this study represent the totality of expression signals that can be extracted from the currently available data, we have named this dataset the *B. subtilis* Modulome.

iModulons derived from different datasets for the same organism can exhibit variability. Such differences arise from variations in experimental conditions, which influence the transcriptomic states captured by the iModulons. For instance, *B. subtilis* iModulons identified from RNA-seq data in this study differ from those obtained from previous microarray datasets. The microarray data included conditions that exposed cells to various DNA-damaging stressors. Consequently, one iModulon captured genes associated with DNA protection and repair, many of which are regulated by LexA. The LexA iModulon showed elevated activities under stress conditions, such as exposure to mitomycin and hydrogen peroxide. In contrast, the RNA-seq dataset revealed the LnrK iModulon, encompassing three genes involved in defense against linearmycins with increased activity upon exposure to the antifungal agent Amphotericin B, suggesting a potential role in resistance to this compound (**Figure K Panels A, B**). Additionally, the MntR iModulon, which includes manganese ABC transporters, was found to be more active in static culture compared to shake culture, corroborating findings from the original study (**Figure K Panels C, D**). These distinct iModulons highlight how variations in growth conditions, environmental factors, and experimental setups can lead to different gene expression patterns, thereby affecting the iModulon structures identified.

Supplementary Figures

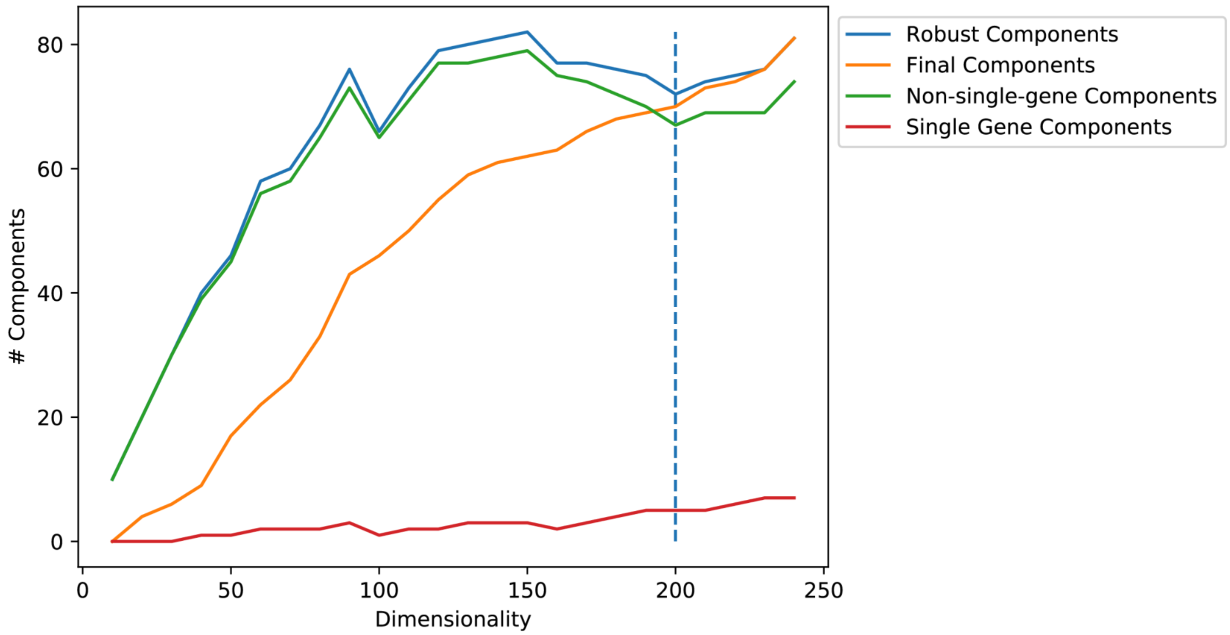

Figure B: Number of components computed at each dimensionality. Robust components are components that are found in over 50% of randomly initialized runs of ICA. Final components are components that have an absolute Pearson correlation coefficient greater than 0.7 with the set of components found at the highest dimension. Single-gene components are components where a single gene dominates the gene coefficients, and non-single-gene components are all components that are not single gene components. The optimal dimensionality is found when the number of non-single-gene components is surpassed by the final components. See McConn et al. [28] for more information.

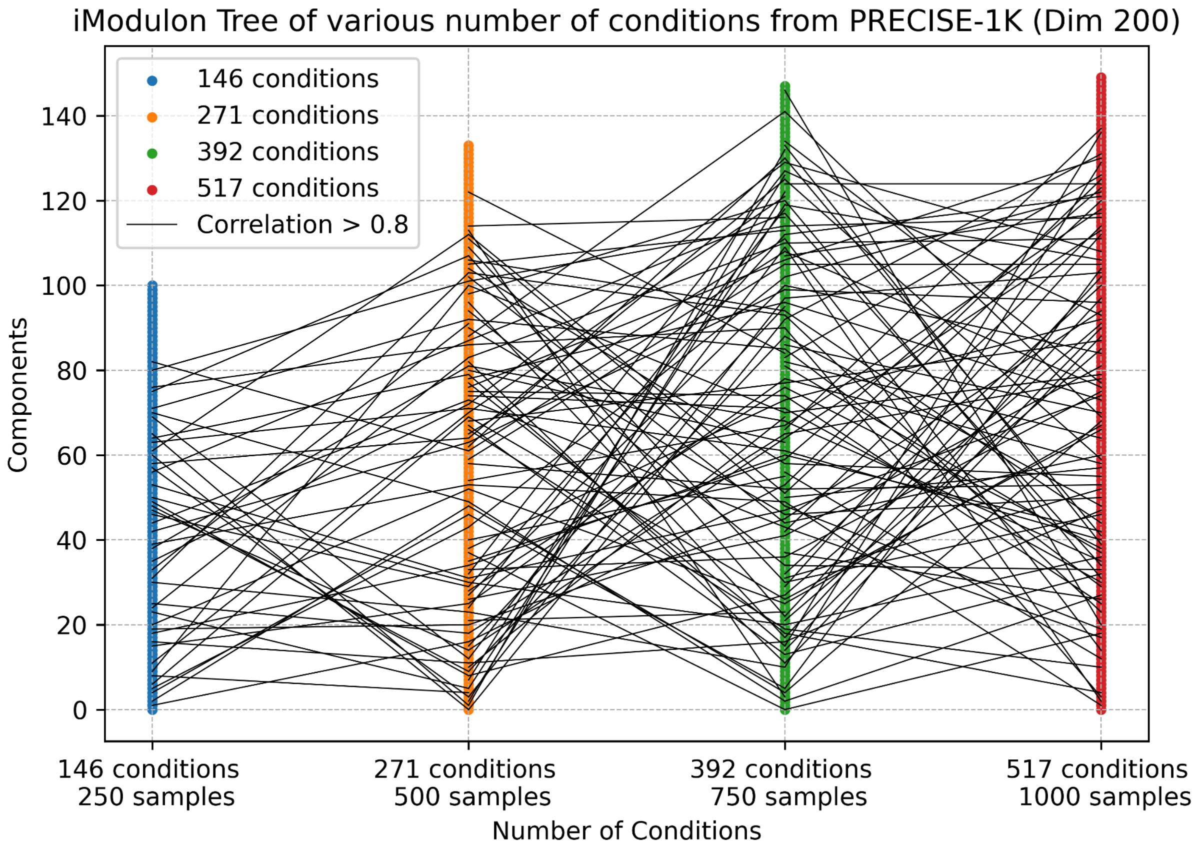

Figure C: iModulon Tree of various number of conditions. Four subsets from PRECISE-1K were analyzed: 146 conditions (250 samples), 271 conditions (500 samples), 392 conditions (750 samples), and 517 conditions (1000 samples). The tree illustrates the changes in iModulons as the number of unique conditions increases.

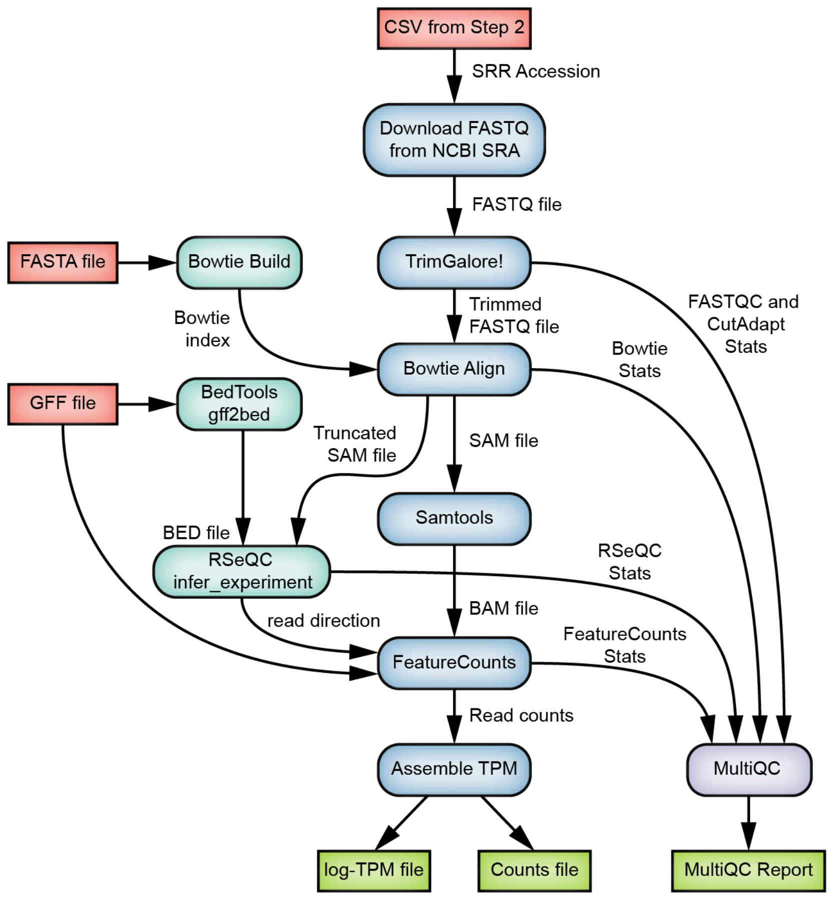

Figure D: Processing pipeline for RNA-seq datasets. Input files are boxed in red, and output files are boxed in green.

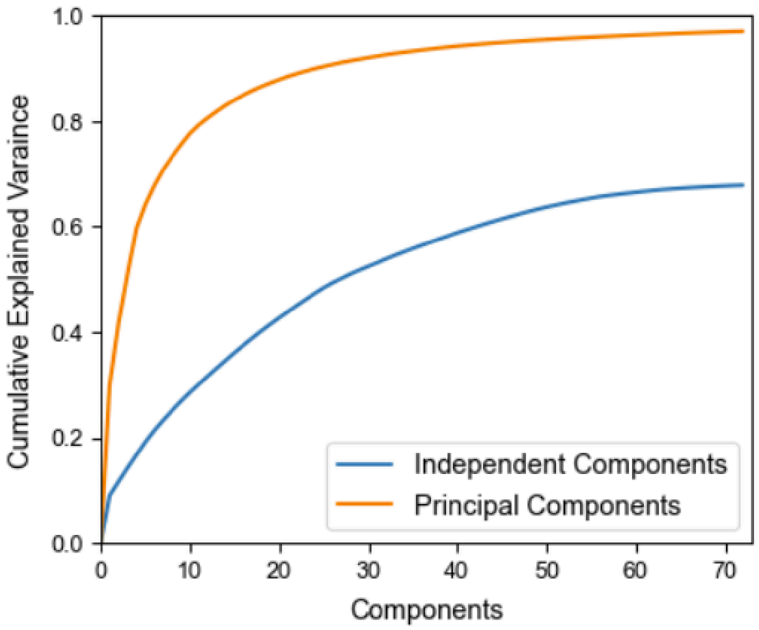

Figure E: Cumulative fraction of expression variance explained by each component for Principal Component Analysis and Independent Component Analysis.

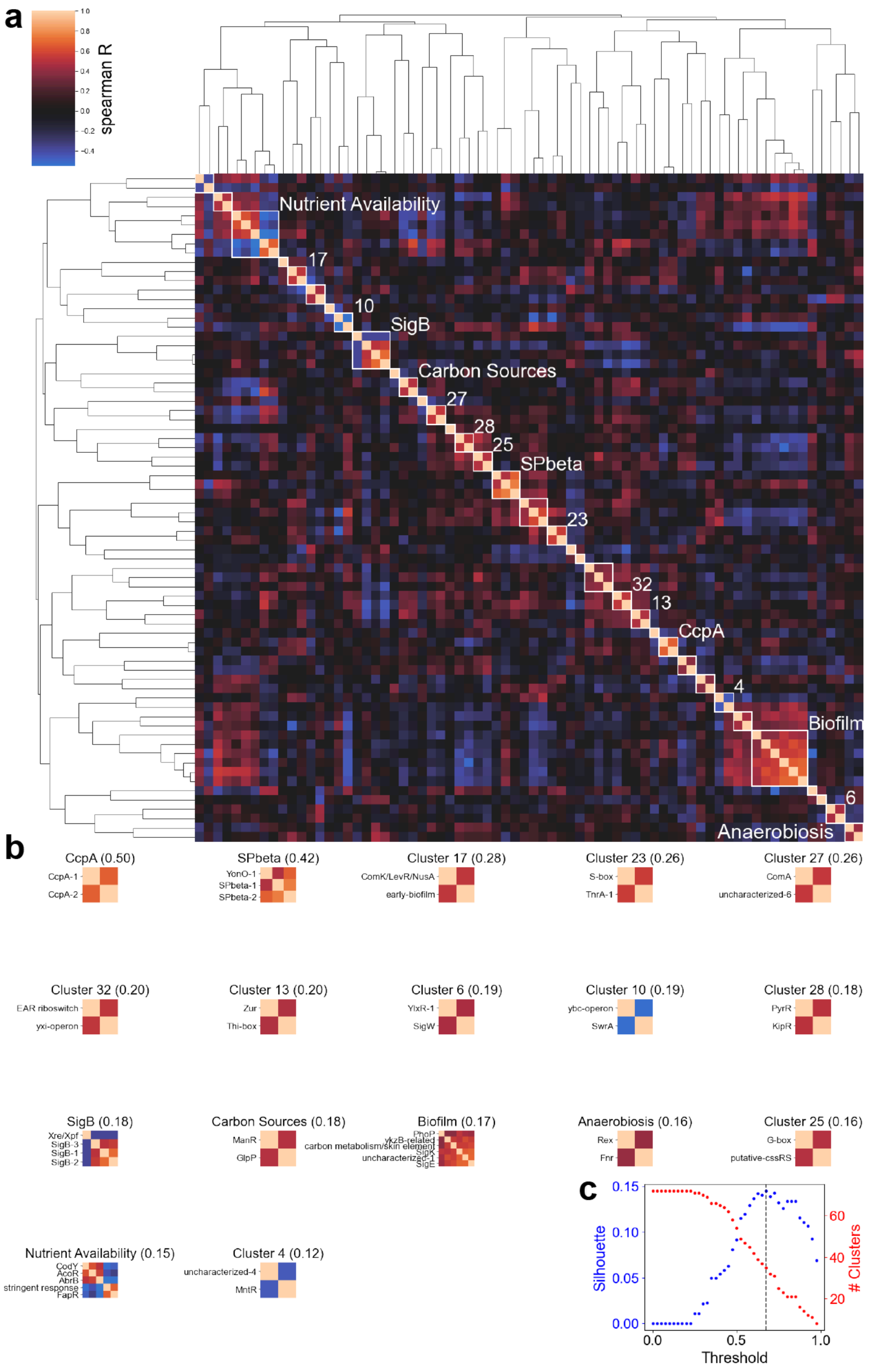

Figure F: (a) Clustered heatmap of Spearman correlations between iModulon activities. (b) Names of iModulons in the best clusters. (c) Sensitivity analysis to identify the clustering threshold.

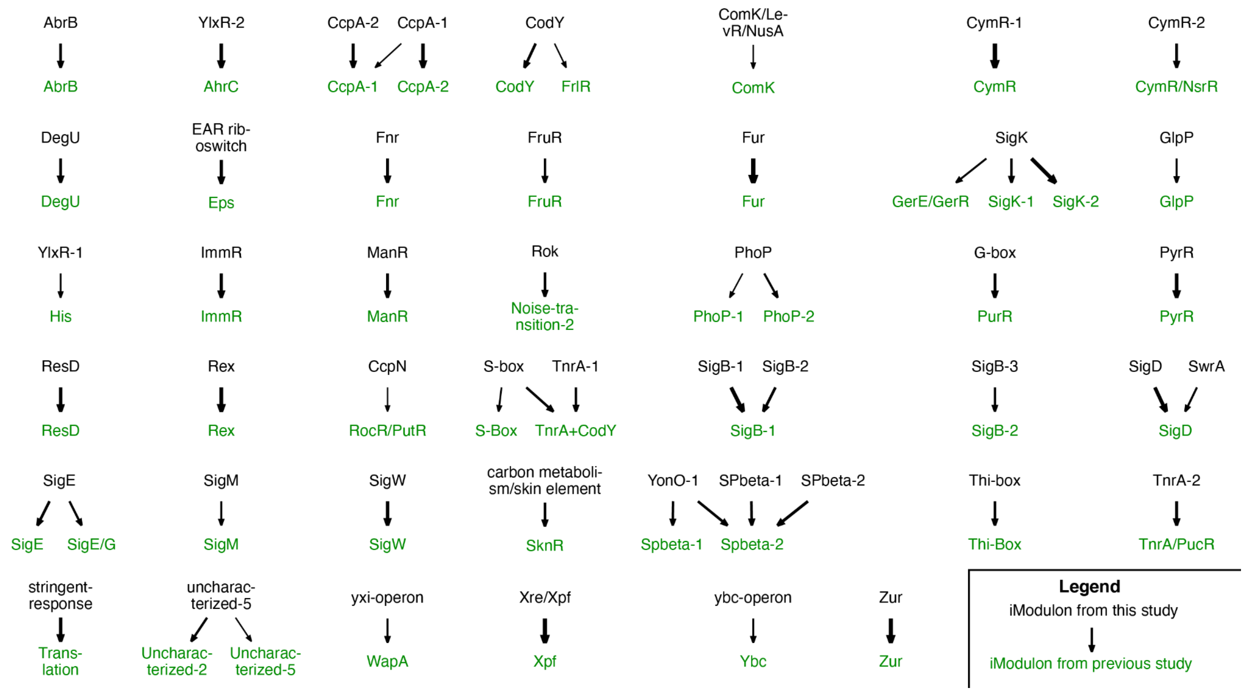

Figure G: Comparison of iModulons computed from the compendium presented in this study (black) against iModulons computed from a microarray dataset (green). Arrow width indicates the Pearson R correlation between the components.

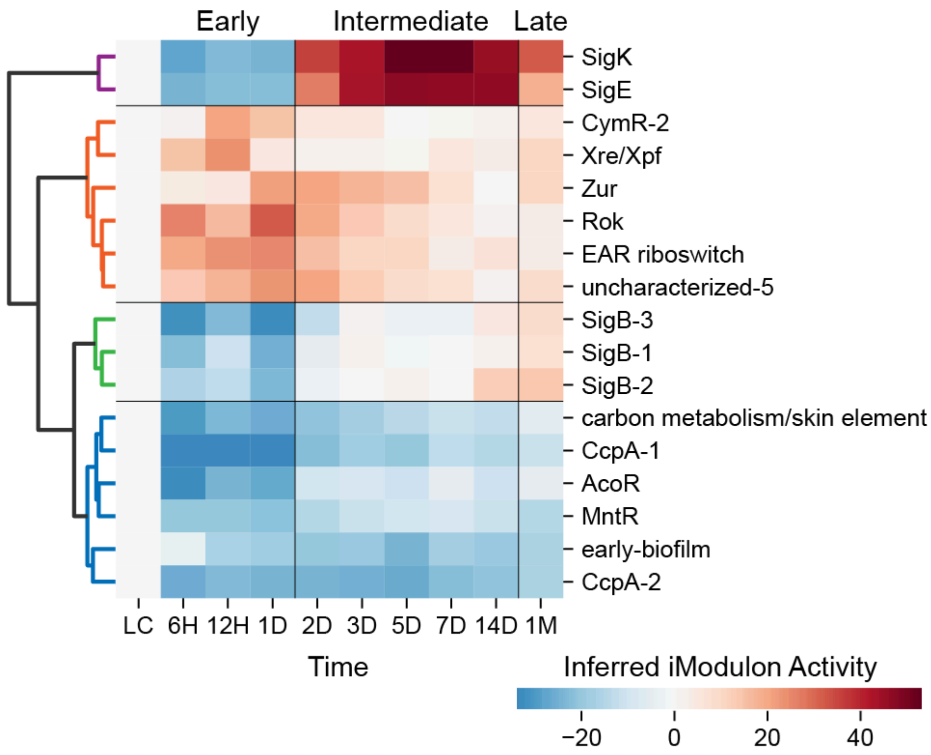

Figure H: Clustermap of inferred iModulon activities across *B. subtilis* biofilm development. Abbreviations: LC - liquid culture, H - hours, D - days, M - month

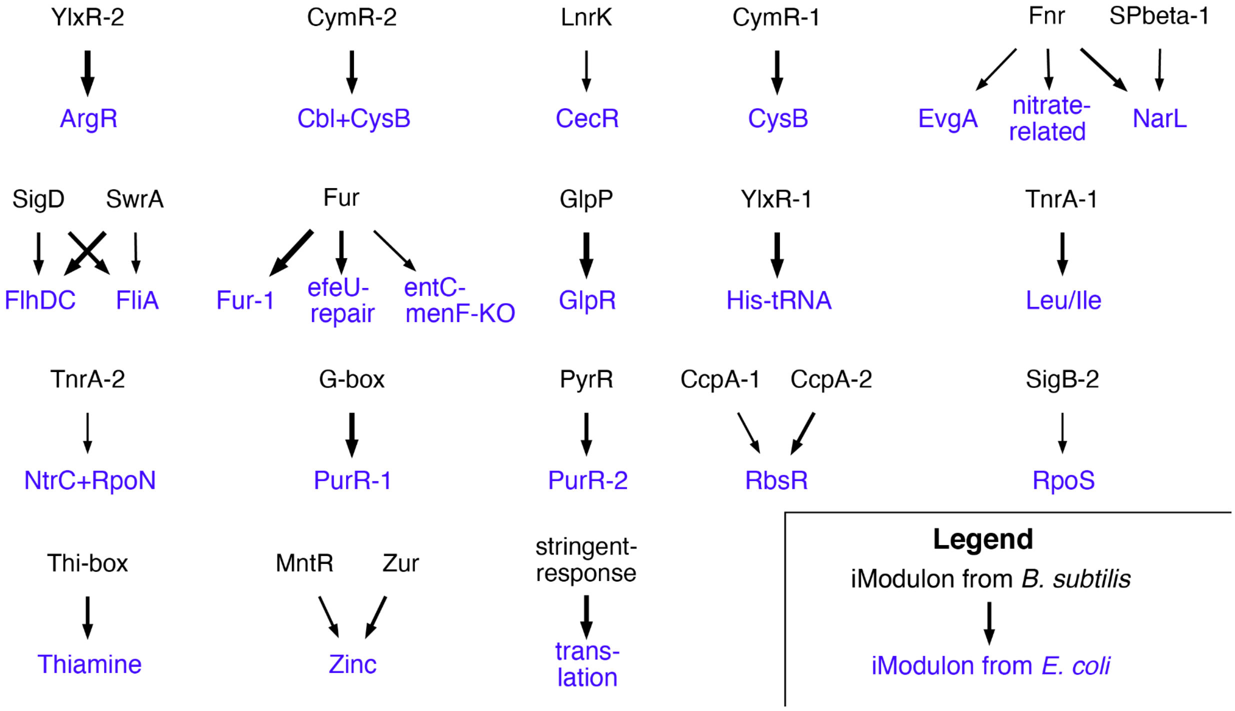

Figure I: Comparison of iModulons computed from the *B. subtilis* RNA-seq compendium presented in this study (black) against iModulons computed from an *E. coli* RNA-seq compendium (blue). Arrow width indicates the Pearson R correlation between the components.

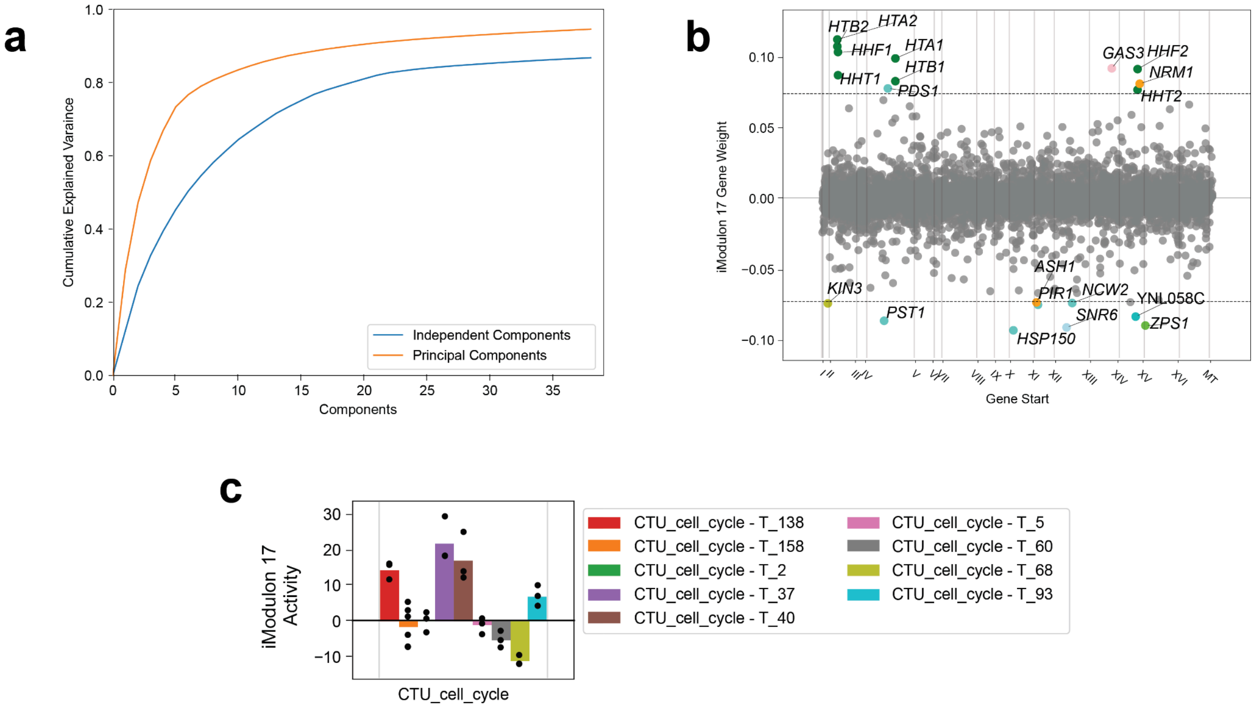

Figure J: Analysis of dataset of *S. cerevisiae* present in the GitHub repository. (A) Cumulative explained variance of each principal component and independent component. (B) Gene weights of iModulon 17 discovered through ICA pipeline. (C) iModulon 17 activities for CTU_cell_cycle project in the dataset.

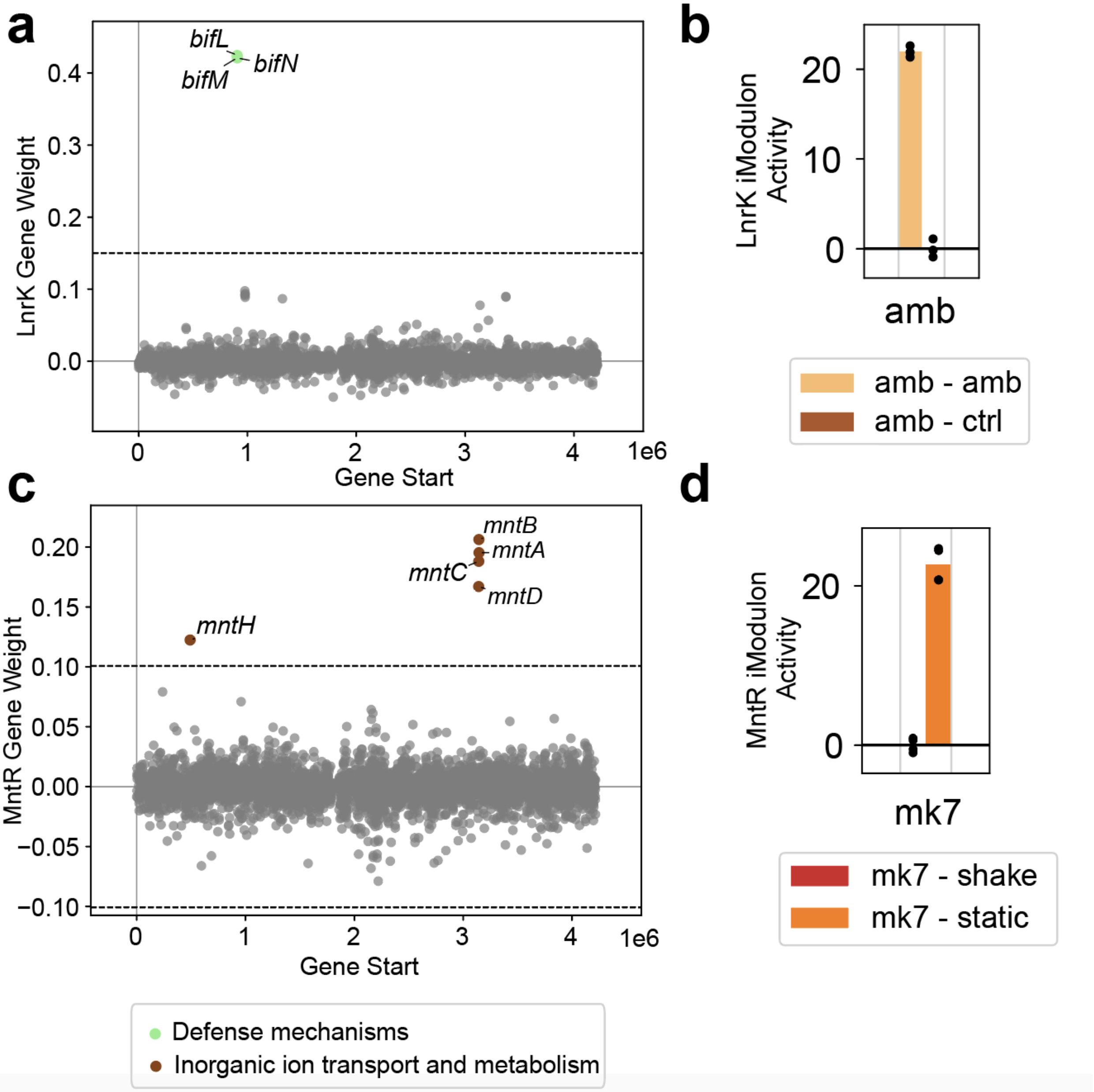

Figure K: Unique iModulons in the *B. subtilis* RNA-seq compendium presented in this study.

Supplementary References

1. Rajesh A, Chang Y, Abedalthagafi MS, Wong-Beringer A, Love MI, Mangul S. Improving the completeness of public metadata accompanying omics studies. Genome Biol. 2021;22: 106.

2. Wang Z, Lachmann A, Ma'ayan A. Mining data and metadata from the gene expression omnibus. Biophys Rev. 2019;11: 103–110.

3. D'agostino RB, Belanger A, D'agostino RB. A Suggestion for Using Powerful and Informative Tests of Normality. Am Stat. 1990;44: 316–321.

4. Pedregosa F, Varoquaux G, Gramfort A, Michel V, Thirion B, Grisel O, et al. Scikit-learn: Machine Learning in Python. J Mach Learn Res. 2011;12: 2825–2830.

5. Kanehisa M, Furumichi M, Sato Y, Ishiguro-Watanabe M, Tanabe M. KEGG: integrating viruses and cellular organisms. Nucleic Acids Res. 2021;49: D545–D551.

6. Huerta-Cepas J, Forslund K, Coelho LP, Szklarczyk D, Jensen LJ, von Mering C, et al. Fast Genome-Wide Functional Annotation through Orthology Assignment by eggNOG-Mapper. Mol Biol Evol. 2017;34: 2115–2122.

7. UniProt Consortium. UniProt: the universal protein knowledgebase in 2021. Nucleic Acids Res. 2021;49: D480–D489.

8. Karp PD, Billington R, Caspi R, Fulcher CA, Latendresse M, Kothari A, et al. The BioCyc collection of microbial genomes and metabolic pathways. Brief Bioinform. 2019;20: 1085–1093.

9. The Gene Ontology Consortium. The Gene Ontology Resource: 20 years and still GOing strong. Nucleic Acids Res. 2019;47: D330–D338.

10. Zhu B, Stülke J. SubtiWiki in 2018: from genes and proteins to functional network annotation of the model organism Bacillus subtilis. Nucleic Acids Res. 2018;46: D743–D748.

11. Bailey TL, Boden M, Buske FA, Frith M, Grant CE, Clementi L, et al. MEME SUITE: tools for motif discovery and searching. Nucleic Acids Res. 2009;37: W202–8.

12. Kılıç S, White ER, Sagitova DM, Cornish JP, Erill I. CollecTF: a database of experimentally validated transcription factor-binding sites in Bacteria. Nucleic Acids Res. 2013;42: D156–D160.

13. Robison K, McGuire AM, Church GM. A comprehensive library of DNA-binding site matrices for 55 proteins applied to the complete Escherichia coli K-12 genome. J Mol Biol. 1998;284: 241–254.

14. Münch R, Hiller K, Barg H, Heldt D, Linz S, Wingender E, et al. PRODORIC: prokaryotic database of gene regulation. Nucleic Acids Res. 2003;31: 266–269.

15. Cipriano MJ, Novichkov PN, Kazakov AE, Rodionov DA, Arkin AP, Gelfand MS, et al. RegTransBase – a database of regulatory sequences and interactions based on literature:

a resource for investigating transcriptional regulation in prokaryotes. BMC Genomics. 2013. p. 213. doi:10.1186/1471-2164-14-213

16. Pachkov M, Balwierz PJ, Arnold P, Ozonov E, van Nimwegen E. SwissRegulon, a database of genome-wide annotations of regulatory sites: recent updates. Nucleic Acids Res. 2013;41: D214–20.
17. Waskom M. seaborn: statistical data visualization. J Open Source Softw. 2021;6: 3021.
18. Rychel K, Decker K, Sastry AV, Phaneuf PV, Poudel S, Palsson BO. iModulonDB: a knowledgebase of microbial transcriptional regulation derived from machine learning. Nucleic Acids Res. 2021;49: D112–D120.
19. IOS Press Ebooks - Jupyter Notebooks – a publishing format for reproducible computational workflows. [cited 12 Mar 2021]. Available: https://ebooks.iospress.nl/publication/42900
20. Santos-Zavaleta A, Salgado H, Gama-Castro S, Sánchez-Pérez M, Gómez-Romero L, Ledezma-Tejeida D, et al. RegulonDB v 10.5: tackling challenges to unify classic and high throughput knowledge of gene regulation in E. coli K-12. Nucleic Acids Res. 2019;47: D212–D220.
21. Escorcia-Rodríguez JM, Tauch A, Freyre-González JA. Abasy Atlas v2.2: The most comprehensive and up-to-date inventory of meta-curated, historical, bacterial regulatory networks, their completeness and system-level characterization. Comput Struct Biotechnol J. 2020;18: 1228–1237.
22. Novichkov PS, Kazakov AE, Ravcheev DA, Leyn SA, Kovaleva GY, Sutormin RA, et al. RegPrecise 3.0--a resource for genome-scale exploration of transcriptional regulation in bacteria. BMC Genomics. 2013;14: 745.
23. Futo M, Opašić L, Koska S, Čorak N, Široki T, Ravikumar V, et al. Embryo-Like Features in Developing Bacillus subtilis Biofilms. Mol Biol Evol. 2021;38: 31–47.
24. Campbell K, Westholm J, Kasvandik S, Di Bartolomeo F, Mormino M, Nielsen J. Building blocks are synthesized on demand during the yeast cell cycle. Proc Natl Acad Sci U S A. 2020;117: 7575–7583.
25. Hyvarinen A. Fast and robust fixed-point algorithms for independent component analysis. IEEE Trans Neural Netw. 1999;10: 626–634.
26. Himberg J, Hyvarinen A. Icasso: software for investigating the reliability of ICA estimates by clustering and visualization. 2003 IEEE XIII Workshop on Neural Networks for Signal Processing (IEEE Cat No03TH8718). IEEE; 2003. pp. 259–268.
27. Sastry AV, Gao Y, Szubin R, Hefner Y, Xu S, Kim D, et al. The Escherichia coli transcriptome mostly consists of independently regulated modules. Nat Commun. 2019;10: 1–14.
28. McConn JL, Lamoureux CR, Poudel S, Palsson BO, Sastry AV. Optimal dimensionality selection for independent component analysis of transcriptomic data. BMC Bioinformatics. 2021;22: 584.
