# Supplementary material for: iModulonMiner and PyModulon: Software for unsupervised mining of gene expression compendia
Source: PLoS Comput Biol. 2024 Oct 23;20(10):e1012546. doi: 10.1371/journal.pcbi.1012546 (PMC11534266; doi:10.1371/journal.pcbi.1012546)
Supplement: Attachment — Submitted filename: reviewer_comment.docx [file pcbi.1012546.s002.docx]

It is clear that Independent Component Analysis (ICA) is a powerful statistical technique that excels in extracting hidden patterns or components from complex data sets. This makes it particularly well-suited for analyzing bulk transcriptome data, which is inherently noisy and high-dimensional, with thousands of genes interacting in complex ways. Because ICA assumes that the observed gene expression data is a linear mixture of independent sources, this model is a reasonable approximation for many biological systems where multiple independent factors can influence gene expression. ICA can disentangle these mixed signals and identify the independent components contributing to the overall gene expression patterns. Furthermore, ICA outperforms WGCNA and biclustering algorithms in detecting known regulons, making it the perfect fit for identifying regulons in complex and noisy RNA-Seq data. It's like finding the right key for the lock. In a series of comprehensive studies on prokaryotic RNA-Seq conducted earlier, Sastry and colleagues demonstrated the detection of iModulons (independently modulated groups of genes) using the ICA algorithm (Menon et al., 2024; Miano et al., 2023; Lamoureux et al., 2023; Rychel et al., 2023; Hirose et al., 2023; Park et al., 2023; Rajput et al., 2022).

In this manuscript, Sastry and colleagues present iModulonMiner, a five-step computational workflow designed for the comprehensive analysis and characterization of RNA-seq data using ICA to obtain independently modulated groups of genes. This workflow begins with the compilation of RNA-seq data from the NCBI SRA database and extends to the analysis and characterization of the totality of RNA-seq data for a given organism or cell type. In this software paper, the authors present PyModulon, a Python package designed to characterize, visualize, and explore computed iModulons. I find this to be a strong and elegant software paper for the following reasons:

- This paper takes, by far, a deep dive into the typical RNA-Seq papers, and this work will be of great significance to transcriptomics studies. Data-driven routine NGS-based transcriptomic studies typically follow a sequence of analysis steps to derive biological insights: differential expression analysis, functional enrichment analysis (GO, KEGG, Reactome), and gene expression/co-expression network analysis. However, given the superior performance of ICA algorithms over methods like WCGNA in identifying gene modules, researchers are increasingly interested in uncovering modules regulated by specific regulatory genes. The availability of software tools like iModulonMiner has facilitated access to this approach for biologists, enabling a deeper understanding of regulatory networks in transcriptomic data (Of course, if sufficient RNA-Seq data provided).
- Furthermore, a critical aspect for end-users in software-focused publications is the availability of comprehensive documentation regarding software usage. Upon examination of the five-step workflow outlined on the GitHub page (https://github.com/SBRG/iModulonMiner), it is evident that the pipeline is well-documented, user-friendly, and easy to navigate. The pipeline's usability would be further enhanced through containerization using Docker.
- Finally, the availability of the Independent Component Analysis of Prokaryotic Transcriptomes (iModulonDB) database (<https://imodulondb.org/index.html>) has greatly enhanced the reproducibility of our research. Despite the increasing availability of RNA-Seq data in the NCBI SRA database and advancements in prokaryotic RNA-Seq algorithms, reproducibility remains a critical concern. To address this, we downloaded approximately 192 *E. coli* RNA-Seq datasets from the NCBI SRA database and successfully processed the resulting count data using iModulonMiner after standard prokaryotic RNA-Seq analysis. Our findings demonstrated a significant overlap with the *E. coli* study results in the iModulonDB database. Notably, we achieved these comparable results despite utilizing our routine analysis pipeline instead of the Nextflow prokaryotic RNA-Seq workflow described in the article. Furthermore, benchmarking different aligners such as Bowtie, Bowtie2, and BWA did not yield substantial outcome differences. (The processed dataset is called the "*E. coli* Modulome" dataset).

Overall, the paper is clear and scientifically correct. I have relatively little suggestion for improvement:

1. The study mentions that the iModulonMiner workflow can be applied to any new organism of interest or cell type, given sufficient RNA-Seq data. However, it is well-established that prokaryotic organisms are simpler than eukaryotic organisms regarding transcriptomic complexity and regulation. Additionally, eukaryotic pathways are often controlled by multiple transcription factors. Therefore, it would be beneficial to demonstrate the applicability of this workflow in a eukaryotic organism, even a simple one like *C. elegans* or *S. cerevisiae*, or to provide relevant references in the manuscript. This would strengthen the claim of the workflow's generalizability and provide valuable insights for researchers working with eukaryotic systems.
2. Downloading RNA-Seq data from the NCBI Sequence Read Archive (SRA) database using tools like esearch or efetch can be quite cumbersome. While manual curation is often cited as the most time-consuming step in research papers, retrieving RNA-Seq data from NCBI can also be a significant bottleneck. To address this, could Aspera Connect software be integrated into scripts to enable faster and, importantly, resumable downloads?
3. I am inquiring for a suggestion only. Can the iModulonMiner workflow be applied to count data obtained from Iso-Seq and Nanopore cDNA sequencing, which are third-generation sequencing methods? (Although limited RNA-Seq data is currently generated with PacBio and Nanopore in the NCBI SRA database, count data can be obtained at the isoform level).
