# Supplementary material for: iModulonMiner and PyModulon: Software for unsupervised mining of gene expression compendia
Source: PLoS Comput Biol. 2024 Oct 23;20(10):e1012546. doi: 10.1371/journal.pcbi.1012546 (PMC11534266; doi:10.1371/journal.pcbi.1012546)
Supplement: Attachment — Submitted filename: Response to Reviewers.docx [file pcbi.1012546.s003.docx]

# Reviewer 1

1. The study mentions that the iModulonMiner workflow can be applied to any new organism of interest or cell type, given sufficient RNA-Seq data. However, it is well-established that prokaryotic organisms are simpler than eukaryotic organisms regarding transcriptomic complexity and regulation. Additionally, eukaryotic pathways are often controlled by multiple transcription factors. Therefore, it would be beneficial to demonstrate the applicability of this workflow in a eukaryotic organism, even a simple one like C. elegans or S. cerevisiae, or to provide relevant references in the manuscript. This would strengthen the claim of the workflow’s generalizability and provide valuable insights for researchers working with eukaryotic systems.

We agree that demonstration in a eukaryotic organism would strengthen our claims of the broad utility of the workflow. Towards addressing this, we note several points. First, we note that ICA was originally demonstrated as a transcription module identification method on eukaryotic data (reviewed here: PMC3005719), and such applications have continued in years since (e.g. PMC2991480, PMC5854612), and thus the ability of ICA to identify transcriptional modules in eukaryotes is supported by the work of other groups as well. Second, we ourselves have recently demonstrated this workflow applied to the yeast *Yarrowia lipolytica* (pre-print here: <https://doi.org/10.1101/2024.07.29.605545>) and are in the process of separately publishing an iModulon analysis for *Saccharomyces cerevisiae*. We now preview some of the results from this analysis in the supplementary work. Additionally, we have now made several improvements to make it clearer to users how the workflow can be applied for eukaryotic organisms. These include updating the iModulonMiner documentation with specific action items throughout the workflow and addition of several functions relevant to this analysis to the PyModulon package which are previewed in the iModulonMiner repository. These include processing of .gff files for yeast and gene weight plotting for visualization of genes on different chromosomes. Additionally, a supplemental figure with preliminary results was added (Figure S9). Functions added to the PyModulon package have been added to the Github repository and are available for download there, and we are currently in the process of updating the PyPI version of the package for pip installation.

2. Downloading RNA-Seq data from the NCBI Sequence Read Archive (SRA) database using tools like esearch or efetch can be quite cumbersome. While manual curation is often cited as the most time-consuming step in research papers, retrieving RNA-Seq data from NCBI can also be a significant bottleneck. To address this, could Aspera Connect software be integrated into scripts to enable faster and, importantly, resumable downloads?

We understand possible concerns about download speed and fidelity. In our experience running ICA on 15+ organisms/databases, the data access step has not been an issue; the first step of the workflow to download the metadata using esearch and efetch typically takes only a few minutes. Among the organisms that we have worked with using iModulons, *E. coli* has the largest dataset. In our most recent download of the metadata for over 15,000 *E. coli* samples, this step took less than 10 minutes to complete. To download and stage the RNA-Seq data, we use fasterq-dump with Nextflow, which processes the download in parallel with other tasks. For datasets with hundreds of samples, the entire Nextflow RNA-Seq processing pipeline only takes a couple of hours. Therefore, we do not expect this step to create bottlenecks in processing time. Thus, we believe additional infrastructure to facilitate data downloads is currently unwarranted, though it may need to be revisited as dataset sizes continue to grow. We have added a comment on the performance of the download step in the new “Workflow computational performance” section, which we added in response to a comment from Reviewer 3.

3. I am inquiring for a suggestion only. Can the iModulonMiner workflow be applied to count data obtained from Iso-Seq and Nanopore cDNA sequencing, which are third-generation sequencing methods? (Although limited RNA-Seq data is currently generated with PacBio and Nanopore in the NCBI SRA database, count data can be obtained at the isoform level).

Although we predominantly run the workflow on TPM normalized data, we have recently tested running on normalized count data, such as that generated by DESeq2 (median of ratios) and TMM normalization. For bacteria, testing on *E. coli* in particular, we recovered nearly identical components, indicating that the workflow can handle count data equally well. Thus, we believe that these newer sequencing datasets will be able to be integrated within existing ICA workflows. However, we were not able to find a sufficiently large Iso-Seq or Nanopore cDNA dataset to attempt running ICA solely on databases containing these data types, in order to determine whether we are able to recover similar components from both sequencing data types. We hope to revisit this issue as more data becomes available. We have now made a comment about the possibilities here in the “Step 2: Processing RNA-seq data” section and **Supplementary Note 1**.

# Reviewer 2

1. What is the effect of the number of varying conditions of organisms on the overall pipeline? Is it possible to demonstrate that with an example and comparison to a baseline?

This is an excellent question that we often ask when analyzing and design datasets for iModulon analysis projects. In partial answer, we show below the effect of sampling subsets of the PRECISE-1K *E. coli* expression database (PMC10602906) on the resulting iModulons. There are two major outcomes. First, the number of components found plateaus starting around 3/4s of the full dataset, indicating that the majority of data added still adds value to the resulting components, but with a limit that we may have recently reached, although it is likely there are still new ‘types’ of conditions still absent from the dataset, such as significant anaerobic or microbial community conditions. Second, even at low dataset numbers, the components that are found are relatively stable. Thus, while smaller datasets may result in an incomplete set of components, they do not appear to result in ‘wrong’ components, i.e. components that would disappear once more data is added. We have now made a mention of these properties in the Results and added details to the Supplementary information (Figure S2).

| Number of samples | 250 | 500 | 750 | 1000 |
| --- | --- | --- | --- | --- |
| Number of conditions | 146 | 271 | 392 | 517 |
| Number of components | 101 | 134 | 148 | 150 |
| Number of conserved components (correlation > 0.8 when comparing to components at 1000 conditions) | 45 | 88 | 117 | 150 |

2. In few of the steps ablations might be possible or be included as option in the pipeline and overall effect may be different. For example, alternatives to ICA (3rd step) and nf-core or STAR (second step) are not explored. Why?

This is an excellent suggestion. As the reviewer states, several alternative methods are possible at certain steps of the workflow. We ourselves have utilized multiple different RNA-Seq processing pipelines and ICA alternatives in a project-specific manner. We have now added a section called “Workflow alternatives” that discusses possible alternatives based on our experience. The ICA step is the core step for iModulons and it can be substituted with other ICA implementations like InfoMax ICA or Picard ICA, as well as sparse ICA. Non-negative Matrix Factorization (NMF) could also be an alternative as described in Fang et al. 2017 (PMID: 28874552). We additionally have added annotations to the Python workflows to indicate where alternative methods could be substituted in. Currently we do not directly implement these alternatives due to the amount of work required to support various alternatives and lack of rigorous comparison of alternatives compared to the primary workflow; we additionally make note of these considerations in the same section.

# Reviewer 3

However, some details are still missing in the manuscript and the github files, making it difficult to repeat the whole calculation procee.

1. The authors stated that in the third step, the data must be inspected to ensure quality, and curated to include all appropriate metadata. This is actually a time-consuming manual work and rely heavily on the expertise of the curator. The authors may provide more detail instructions on how to do it properly to ensure the reliability of the calculated modulons. They may add the instructions in the B. subtilis results section so that readers can actually follow the steps to perform the analysis using the B. subtilis data.

We thank the reviewer for this suggestion. The instructions and recommendations for metadata curation were previously included at the end of the expression_QC_part1.ipynb, with an example metadata sheet provided in the GitHub repository. We have now added additional sentences in the manuscript to guide the reader to these instructions, as follows:

Design and Implementation Section - Step 3: Quality Control and Data Normalization

[... Manual curation of metadata is also performed to include experimental details of the samples. These include the strain of the sample, culture media, temperature, growth phase, and relevant experimental information that can facilitate downstream iModulon characterization. The recommended curations can be found in the step3 instructions (https://github.com/SBRG/iModulonMiner/blob/main/3_quality_control/expression_QC_part1.ipynb). …]

2. More data should be added for the pipeline such as the calculation time in a typical computer and the data and file sizes at each step.

We thank the reviewer for this excellent suggestion. We show the results of this analysis below on the PRECISE-1K *E. coli* RNA-Seq compendium consisting of 4257 genes and 1035 samples, with an input gene expression log2(TPM) matrix of 80.2 MB. The machine used has the following specs:

CPU: AMD Ryzen Threadripper PRO 5995WX (256 MB cache, 64 cores, 128 threads)

RAM: 256GB, DDR4, 3200 MT/s

64 threads were used for the following evaluation

Time at different dimensionalities for the optICA calculation:

|  | 100 FastICA runs  (Total) | Distance Matrix & Clustering | Gathering final  matrices |
| --- | --- | --- | --- |
| Dim 50 | 22.71 seconds | 26.15 seconds | 4.68 seconds |
| Dim 100 | 57.13 seconds | 37.32 seconds | 8.63 seconds |
| Dim 150 | 3.34 minutes | 52.69 seconds | 13.93 seconds |
| Dim 200 | 7.96 minutes | 1.10 minutes | 16.02 seconds |
| Dim 250 | 15.47 minutes | 1.13 minutes | 20.76 seconds |
| Dim 300 | 25.12 minutes | 1.14 minutes | 24.50 seconds |

Resulting file sizes:

|  | Temporary files | Final M matrix | Final A matrix |
| --- | --- | --- | --- |
| Dim 50 | 543 MB | 4.4 MB | 970.8KB |
| Dim 100 | 1.1 GB | 8.2 MB | 1.8 MB |
| Dim 150 | 1.6 GB | 11.4 MB | 2.4 MB |
| Dim 200 | 2.2 GB | 13.3 MB | 2.9 MB |
| Dim 250 | 2.6 GB | 15.4 MB | 3.3 MB |
| Dim 300 | 3.3 GB | 18 MB | 3.9 MB |

We have added this information to the Design and Implementation section and **Supplementary Note 3** so that users can anticipate the computational requirements.

3. I have tried to use the PyModulon to repeat the calculations but failed at step 2. I always got the “nextflow: command not found” error message when I run “nextflow run [main.nf](http://main.nf/) -profile local --organism bacillus_subtilis --metadata ../test/test_metadata.tsv --sequence_dir ../test/sequence_files/ --outdir ../test/nf_results/” command. Even though they said “install the dependencies in requirements.txt” in the github readme file, I actually could not find the requirements.txt file in the iModulonMiner project. The file required for generating Gene table and KEGG/GO annotations, “gene_annotation.ipynb”, is also missing.

We thank the reviewer for bringing this to our attention. This seems to be an issue due to a file inappropriately retaining an old name from a previous fork. We had changed the “requirement.txt” file to the environment.yml file, but forgot to reflect this change in the README files. We have corrected this error in the README files for Steps 3 and 5, as well as the folder ‘figures’. The “environment.yml” file includes the requirement for nextflow that is needed to run Step 2. The nextflow requirements can also be found in the “nextflow_environment.yml” file in the 2_process_data folder. These instructions are also updated in the README files.

The gene_annotation.ipynb was renamed to 1_create_the_gene_table.ipynb in the folder ‘5_characterize_iModulons’. We have corrected the reference in Step 5, 2_iModulon_characterization.ipynb to reflect this change.

4. More discussion on the comparison of the 72 imodulons obtained from the new pipeline and the 83 imodulons from their previous study (ref 10) should be added. The manuscript mentioned that 47 iModulons (65%) were highly similar but how the similarity was quantitatively defined was not provided. I am not sure if this number is enough to prove the calculation results is robust. There is no discussion on the 25 imodulons (36 from previous study) which are not similar. The author may need to provide the complete data in a supplementary file for further biological examination.

Thank you for the comments and suggestions. The similarities between the 47 iModulons and iModulons from previous runs are defined by the Pearson R correlation between the independent component gene weights. The correlation coefficients are represented by the thickness of the arrows Figure S6. We have added the following sentence to clarify this:

Results section - Comparing iModulon structures across datasets and organisms reveals robustness

[... The similarities are defined by the Pearson R correlation between the independent component gene weights, and are represented by the thickness of the arrows in ~~(~~**Figure S6**~~)~~. …]

We also provided more investigations on the iModulons that are dissimilar between the two runs in the supplements.

Results section - Comparing iModulon structures across datasets and organisms reveals robustness

[... The differences in dataset and experimental conditions can create different iModulons within the same organism (see Supplementary Note 5), but …]

Supplementary Note 5 - Analyzing iModulon differences across Datasets

…

iModulons derived from different datasets for the same organism can exhibit variability. Such differences arise from variations in experimental conditions, which influence the transcriptomic states captured by the iModulons. For instance, iModulons identified from RNA-seq data in this study differ from those obtained from previous microarray datasets. The microarray data included conditions that exposed cells to various DNA-damaging stressors. Consequently, one iModulon captured genes associated with DNA protection and repair, many of which are regulated by LexA. The LexA iModulon showed elevated activities under stress conditions, such as exposure to mitomycin and hydrogen peroxide. In contrast, the RNA-seq dataset revealed the LnrK iModulon, encompassing three genes involved in defense against linearmycins with increased activity upon exposure to the antifungal agent Amphotericin B, suggesting a potential role in resistance to this compound (**Figure S10a, b**). Additionally, the MntR iModulon, which includes manganese ABC transporters, was found to be more active in static culture compared to shake culture, corroborating findings from the original study (**Figure S10c, d**). These distinct iModulons highlight how variations in growth conditions, environmental factors, and experimental setups can lead to different gene expression patterns, thereby affecting the iModulon structures identified.
